# Impact of blood urea nitrogen to creatinine ratio on mortality and morbidity in hemodialysis patients: The Q-Cohort Study

**DOI:** 10.1038/s41598-017-14205-2

**Published:** 2017-11-02

**Authors:** Shigeru Tanaka, Toshiharu Ninomiya, Masatomo Taniguchi, Masanori Tokumoto, Kosuke Masutani, Hiroaki Ooboshi, Takanari Kitazono, Kazuhiko Tsuruya

**Affiliations:** 1 Division of Internal Medicine, Fukuoka Dental College, Fukuoka, Japan; 2 Department of Epidemiology and Public Health, Graduate School of Medical Sciences, Kyushu University, Fukuoka, Japan; 3 Fukuoka Renal Clinic, Fukuoka, Japan; 4 Department of Medicine and Clinical Science, Graduate School of Medical Sciences, Kyushu University, Fukuoka, Japan; 5 Department of Integrated Therapy for Chronic Kidney Disease, Graduate School of Medical Sciences, Kyushu University, Fukuoka, Japan

## Abstract

The association between blood urea nitrogen to creatinine ratio (UCR) and survival is uncertain in hemodialysis patients. We examined the influence of UCR on mortality and morbidity in hemodialysis patients. A total of 3,401 hemodialysis patients were prospectively followed for 4 years. The association between UCR with overall survival was analyzed using a Cox regression model. During a 4-year follow-up period, 545 patients died from any cause and 582 experienced MACE, 392 with coronary heart disease (CHD), 114 with infection-related death, 77 with hemorrhagic stroke, 141 with ischemic stroke, and 107 with cancer death. Every 1 increase in UCR level was significantly associated with an increased risk for all-cause mortality (hazard ratio [HR] 1.07; 95% confidence interval [CI] 1.03–1.12), CHD (HR 1.08; 95% CI 1.02–1.14), and infection-related death (HR 1.11; 95% CI 1.02–1.21). There was no evidence of a significant association between UCR and death from cancer, and incidence of stroke. A high UCR was significantly associated with an increased risk for all-cause mortality, infection-related death and incidence of CHD in hemodialysis patients.

## Introduction

Protein-energy malnutrition (PEM) and inflammation are generally concomitant in maintenance hemodialysis patients. Mutual interaction between these conditions, so-called malnutrition-inflammation complex syndrome (MICS), is well established as a powerful indicator of a poor prognosis in hemodialysis patients^1,2^. Although the mechanisms causing MICS are complex and not fully determined, it is assumed that a vicious cycle formed by uremia-induced abnormalities in protein metabolism^3^, systemic chronic inflammation^4,5^, and increased oxidative stress^6,7^ might contribute to sarcopenia status, resulting in an increased risk for cardiovascular disease (CVD) and infection in hemodialysis patients. Establishment of a concise and discriminative screening marker to detect MICS is required for dialysis management, with the aim of improving life expectancy.

Recently, the blood urea nitrogen to creatinine ratio (UCR) has emerged as an independent predictor of poor clinical outcomes in various population settings, such as acute kidney injury^8^, chronic heart failure^9–11^, and ischemic stroke^12^. Epidemiological evidence suggests that UCR could reflect dietary protein intake at each level of renal function^13–15^. Additionally, UCR increases greatly after episodes of inter-current illness, such as infections and other catabolic stresses, under controlled dietary conditions^14^. These findings suggest that this ratio might be a potential indicator of an increased catabolic burden generally coexisting with PEM and inflammation.

Although it has been reported that there is a significant association between elevated UCR and an increased risk for early death in hemodialysis patients^16,17^, the precise relationship between UCR and risk for mortality and morbidity remains uncertain. We hypothesize that UCR is a marker reflecting increased catabolic burden and inflammation and may be useful for detecting risks of potential PEM and MICS in hemodialysis patients. In this study, we assessed the clinical utility of UCR in predicting mortality and morbidity in a large-scale, prospective cohort of hemodialysis patients.

## Methods

### Study population

A detailed description of the survey design of the Q-Cohort Study has been previously described^18^. Briefly, a total of 3,598 outpatients aged 18 years or older undergoing hemodialysis at 39 dialysis facilities in the Saga and Fukuoka prefectures, in the northern region of Kyushu Island, Japan were registered between 31 December 2006 and 31 December 2007. Sixty-six patients who had missing data on one or more clinical variables, two patients whose UCR value was extremely high (>25), 32 patients who showed extreme outliers in C-reactive protein (greater than the 99th percentile; 5.79 mg/dL), and 97 patients whose information of outcome could not be obtained were excluded. Finally, the remaining 3,401 patients were enrolled in the current study. Dialysis period of included patients was less than 1 month at the longest to the longest 40.6 years.

The study was conducted with the approval of the Clinical Research Ethics Committee of the Institutional Review Board at Kyushu University (Approval Number 20-31) and all participating institutions. Written informed consent was obtained from all participants. We confirm that all methods were performed in accordance with the relevant guidelines and regulations. The study is registered at the University Hospital Medical Information Network (UMIN) clinical trial registry (UMIN000000556). Patients were followed prospectively from the date that each patient was registered to participate in the study until 31 December 2010.

### Covariates

The main exposure was blood UCR level at baseline. Data on baseline characteristics and potential confounders (age, sex, dialysis vintage, presence of diabetes mellitus, history of CVD, pre-dialysis systolic blood pressure, pre-dialysis diastolic blood pressure, serum level of albumin, corrected calcium (Ca), phosphorus, and C-reactive protein, Kt/V, normalized protein catabolic rate [nPCR], body mass index [BMI], and use of antihypertensive agents) were collected by reviewing medical records. Blood samples were collected before starting dialysis. Serum concentrations of albumin, Ca, phosphorus, alkaline phosphatase, and parathyroid hormone were determined using standard methods^18^. The corrected serum Ca concentration was calculated depending on the serum albumin concentration based on Payne’s formula; corrected Ca (mg/dL) = observed total Ca (mg/dL) + (4.0 − serum albumin concentration [g/dL]).

### Outcomes

The primary outcome was the all-cause mortality rate and secondary outcomes were the incidence of infection-related death, cancer mortality, and major cardiovascular events (MACE), which was defined as a first-ever development of cardiovascular death, stroke, myocardial infarction, hospitalization for unstable angina, coronary intervention (coronary artery bypass surgery or angioplasty), hospitalization for heart failure, and/or peripheral vascular disease^18^. The health status of participants was checked annually by local physicians at each dialysis facility and by mail or telephone for any patient who moved to another dialysis facility where a collaborator of this study was not present. Death events were determined on the basis of patients’ medical records.

### Statistical analysis

Blood UCR levels were divided into quartile groups (<5.35, 5.36–6.44, 6.45–7.70, and >7.70). Differences in continuous variables were determined using the Kruskal–Wallis one-way test. Categorical variables were compared using the Fisher’s exact test. Since results from the initial exploration for the primary outcome (all-cause mortality) revealed a potentially nonlinear relationship between UCR and morality, we analyzed UCR as a continuous variable fitting a restricted cubic spline model with four knots, which were placed at the recommended 5th, 35th, 65th, and 95th percentiles of UCR^19^. Median UCR level (6.44) was selected as the reference value for all spline plots. Age- and sex-adjusted and multivariate-adjusted hazard ratios (HR) with 95% confidence intervals (95% CI) of each risk factor for the development of events were calculated using a Cox proportional hazards model. The second quartile (5.36–6.44) was selected as the reference group because restricted cubic spline analysis showed that this range was associated with the lowest risk for all-cause mortality. All models were adjusted for potential risk factors for cardiovascular outcomes: age, sex, dialysis vintage, presence of diabetes mellitus, history of CVD, pre-dialysis systolic blood pressure, serum levels of albumin, corrected Ca, phosphorus, total cholesterol, and C-reactive protein, Kt/V, nPCR, BMI, and use of antihypertensive agents. We assessed the comparative predictability for all-cause mortality using Harrell C statistics between models incorporating UCR or other protein catabolic markers. Harrell C statistics and 95% CI were calculated and compared using the somersd package and lincom commands, respectively^20^. In addition, predictive performance for future mortality risk with or without inclusion of UCR was evaluated by the net reclassification improvement (NRI) and the integrated discrimination improvement (IDI)^21^. The cutoff values of reclassification were determined according to tertile group of predicted probabilities of all-cause death incidence (<5%, 5–15%, and ≥15%). Statistical analyses were conducted using the SAS software package (ver. 9.3; SAS Institute, Cary, NC) and the STATA software package (ver. 14.0; Stata Corp., College Station, TX). A two-tailed P-value < 0.05 was considered statistically significant.

## Results

**Figure 1:**
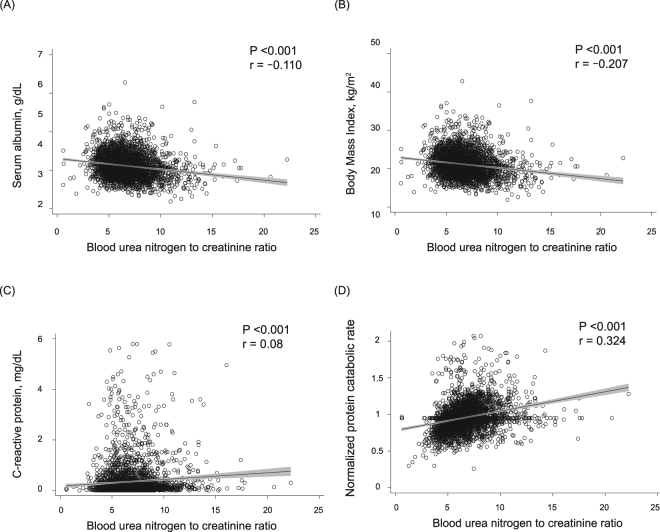
Relationships between blood urea nitrogen to creatinine ratio (UCR) and serum albumin (**A**), body mass index (BMI) (**B**), C-reactive protein (**C**), or normalized protein catabolic rate (nPCR) (**D**) in the prospective longitudinal cohort (n = 3,401).

At baseline, a total of 3598 enrolled dialysis patients, 3,401 participants (94.5%) were evaluable. The median follow-up period was 48 months (25–75th percentiles, 33.5–48 months). The overall mean ± standard deviation of blood UCR level was 6.7 ± 2.0. Baseline characteristics of all participants in the cohort are shown in Table 1. Patients with a higher UCR were older, more likely to be female and have a shorter dialysis duration, and had a greater frequency of diabetes and history of CVD. Mean values of diastolic blood pressure, serum albumin, serum corrected Ca, and BMI decreased with increasing UCR levels. In contrast, significant upward trends were observed in mean values for serum total cholesterol, serum C-reactive protein, Kt/V, and nPCR with higher UCR levels. Figure 1 shows significant negative correlations between UCR and nutritional indicators (*r* = −0.110, *P* < 0.001 for albumin and *r* = −0.207, *P* < 0.001 for BMI) and positive correlations for C-reactive protein *(r* = 0.08, *P* < 0.001) and nPCR (*r* = 0.324, *P* < 0.001).

**Table 1:** Baseline characteristics of participants in each group divided by UCR level.

	All patients (n = 3,401)	Categories of blood UCR level				P-value
		≤5.35 (n = 852)	5.36–6.44 (n = 846)	6.45–7.70 (n = 850)	>7.70 (n = 853)	
Age (years)	63.6 (12.8)	59.4 (13.1)	61.6 (12.6)	64.6 (11.9)	68.9 (10.3)	<0.001
Gender (male) (%)	59.2	79.8	68.0	53.9	35.2	<0.001
Dialysis vintage (years)	5.5 (2.1–11.5)	7.0 (3.5–12.6)	6.5 (2.8–11.9)	5.3 (1.9–12.4)	3.4 (1.0–8.7)	<0.001
Diabetes mellitus (%)	28.9	22.0	27.4	29.7	36.7	<0.001
History of cardiovascular disease (%)	22.5	18.1	21.9	21.2	29.0	<0.001
Pre-dialysis systolic blood pressure (mmHg)	153.1 (23.3)	152.3 (22.8)	153.1 (22.5)	153.8 (23.6)	153.3 (24.4)	0.79
Pre-dialysis diastolic blood pressure (mmHg)	76.4 (12.5)	78.7 (12.8)	77.0 (12.8)	75.9 (11.9)	74.2 (12.1)	<0.001
Serum albumin (g/dL)	3.8 (0.4)	3.8 (0.5)	3.9 (0.4)	3.8 (0.4)	3.7 (0.5)	<0.001
Serum corrected calcium (mg/dL)	9.4 (0.8)	9.5 (0.8)	9.4 (0.7)	9.3 (0.7)	9.3 (0.7)	<0.001
Serum phosphorus (mg/dL)	4.9 (1.2)	4.8 (1.2)	5.0 (1.2)	5.0 (1.2)	5.0 (1.2)	0.002
Serum total cholesterol (mg/dL)	155.9 (36.7)	148.9 (35.9)	153.8 (33.9)	157.7 (35.7)	163.1 (39.3)	<0.001
Serum C-reactive protein (mg/dL)	0.35 (0.66)	0.31 (0.6)	0.33 (0.9)	0.32 (0.6)	0.42 (0.8)	<0.001
Kt/V (single pool)	1.56 (0.3)	1.51 (0.2)	1.56 (0.3)	1.61 (0.3)	1.63 (0.3)	<0.001
nPCR (g/kg/day)	0.96 (0.2)	0.87 (0.2)	0.95 (0.2)	0.99 (0.2)	1.0 (0.2)	<0.001
Body mass index (kg/m^2^)	21.2 (3.1)	21.8 (3.0)	21.5 (2.9)	20.9 (3.0)	20.4 (3.4)	<0.001
Use of antihypertensive agents (%)	62.6	62.6	60.9	64.4	62.6	0.53

*Note:* Continuous data expressed as mean ± standard deviation; categorical data, as percentages. Dialysis vintage is shown as the median (interquartile range). Abbreviations: UCR; urea nitrogen to creatinine ratio, nPCR; normalized protein catabolic rate.

Multivariable Cox proportional hazard models showed that serum high UCR levels were independently associated with an increased risk for all-cause mortality, CHD, and infection-related death (Table 2). After adjusting for the above-mentioned confounding factors, every 1.0 increase in UCR was associated with a 1.07-fold (95% CI 1.03–1.12) increased risk for all-cause mortality. With regard to the incidence of CHD and infection-related death, the multivariable-adjusted risk per 1.0 increase in UCR also increased significantly, by 1.08-fold (95% CI 1.02–1.14) for CHD and 1.11-fold (95% CI 1.02–1.21) for infection-related death (Table 2).

**Table 2:** Associations between UCR levels and risk for mortality and morbidity.

Model	HR (95% CI) by UCR level				
	UCR per 1 increment (n = 3,401)	≤5.35 (n = 852)	5.36–6.44 (n = 846)	6.45–7.70 (n = 850)	>7.70 (n = 853)
All-cause death, No. (%)	545 (16.0)	113 (13.3)	104 (12.3)	114 (13.4)	214 (25.1)
Age- and sex-adjusted	1.11 (1.07–1.15)*	1.19 (0.91–1.55)	1 (reference)	1.02 (0.78–1.33)	1.80 (1.40–2.30)*
Multivariable-adjusted^a^	1.07 (1.03–1.12)*	1.09 (0.83–1.43)	1 (reference)	0.98 (0.75–1.29)	1.55 (1.20–2.00)*
MACE, No. (%)	582 (17.1)	132 (15.5)	136 (16.1)	139 (16.4)	175 (20.5)
Age- and sex-adjusted	1.07 (1.03–1.12)*	0.99 (0.78–1.26)	1 (reference)	1.03 (0.81–1.30)	1.34 (1.06–1.70)*
Multivariable-adjusted^a^	1.04 (0.99–1.09)	1.07 (0.83–1.36)	1 (reference)	1.03 (0.81–1.31)	1.21 (0.95–1.55)
CHD, No. (%)	392 (11.5)	88 (10.3)	89 (10.6)	91 (10.7)	124 (14.5)
Age- and sex-adjusted	1.11 (1.05–1.16)*	1.00 (0.74–1.35)	1 (reference)	1.03 (0.77–1.38)	1.50 (1.12–2.00)*
Multivariable-adjusted^a^	1.08 (1.02–1.14)*	1.08 (0.80–1.46)	1 (reference)	1.04 (0.78–1.40)	1.38 (1.03–1.85)*
Infection-related death, No. (%)	114 (3.4)	22 (2.6)	17 (2.0)	19 (2.2)	56 (6.6)
Age- and sex-adjusted	1.17 (1.09–1.26)*	1.41 (0.75–2.66)	1 (reference)	1.02 (0.53–1.97)	2.80 (1.58–4.93)*
Multivariable-adjusted^a^	1.11 (1.02–1.21)*	1.24 (0.65–2.36)	1 (reference)	0.89 (0.46–1.74)	2.10 (1.17–3.77)*
Hemorrhagic stroke, No. (%)	77 (2.3)	20 (2.4)	22 (2.6)	19 (2.2)	16 (1.9)
Age- and sex-adjusted	1.02 (0.90–1.17)	0.86 (0.47–1.59)	1 (reference)	0.98 (0.53–1.82)	0.99 (0.51–1.95)
Multivariable-adjusted^a^	0.98 (0.84–1.13)	0.97 (0.52–1.81)	1 (reference)	0.92 (0.49–1.71)	0.90 (0.44–1.81)
Ischemic stroke, No. (%)	141 (4.2)	32 (3.8)	30 (3.6)	38 (4.5)	41 (4.8)
Age- and sex-adjusted	0.98 (0.90–1.08)	1.17 (0.71–1.93)	1 (reference)	1.18 (0.73–1.92)	1.19 (0.72–1.95)
Multivariable-adjusted^a^	0.95 (0.86–1.05)	1.16 (0.70–1.93)	1 (reference)	1.14 (0.70–1.86)	1.04 (0.63–1.74)
Cancer death, No. (%)	107 (3.2)	25 (2.9)	29 (3.4)	25 (2.9)	28 (3.3)
Age- and sex-adjusted	0.94 (0.85–1.05)	0.96 (0.56–1.64)	1 (reference)	0.76 (0.44–1.30)	0.74 (0.43–1.28)
Multivariable-adjusted^a^	0.97 (0.87–1.08)	0.78 (0.45–1.36)	1 (reference)	0.80 (0.46–1.38)	0.78 (0.44–1.36)

*P < 0.05. Abbreviations: MACE, major cardiovascular events; CHD, coronary heart disease; HR, hazard ratio; CI, confidence interval. ^a^Adjusted for baseline characteristics (age, sex, dialysis vintage, diabetes, history of cardiovascular disease, pre-dialysis systolic blood pressure, serum levels of albumin, corrected calcium, phosphorus, total cholesterol, and log-transformed C-reactive protein, Kt/V, normalized protein catabolic rate, body mass index, and antihypertensive agent use).

### All-cause mortality

During the follow-up period, 545 (16.0%) patients died from any causes. Compared with the reference UCR group of 5.36–6.44 (13.3% with all-cause death), the highest UCR (25.1%; [HR 1.80; 95% CI, 1.40–2.30] for >7.70) was associated with an increased risk for all-cause death after adjustment for age and sex (Table 2). This relationship remained substantially unchanged after adjustment for potential confounding factors (HR 1.55; 95% CI 1.20–2.00 for >7.70).

### MACE and CHD

MACE occurred in 582 (17.1%) participants and CHD occurred in 392 (11.5%). Compared with the reference UCR group of 5.36–6.44 (16.1% with MACE and 10.6% with CHD), the highest UCR (20.5% with MACE and 14.5% with CHD) was significantly associated with an increased risk for MACE (HR 1.34; 95% CI 1.06–1.70 for >7.70) and CHD (HR 1.50; 95% CI, 1.12–2.00 for >7.70) after adjustment for age and sex. There was a significant association between UCR categories and incidence risk for CHD (HR 1.38; 95% CI 1.03–1.85 for >7.70), but not for MACE (HR 1.21; 95% CI 0.95–1.55 for >7.70) after adjustment for potential confounding factors.

### Infection-related death

Infection-related death occurred in 114 (3.4%) participants. Compared with the reference UCR group of 5.36–6.44 (2.0% with infection-related death), the highest UCR (6.6% with infection-related death) was associated with an increased risk for infection-related death (HR 2.80; 95% CI 1.58–4.93 for >7.70) after adjustment for age and sex. This relationship did not change after adjustment for potential confounding factors (HR 2.10; 95% CI 1.17–3.77 for >7.70).

### Hemorrhagic and ischemic stroke

Hemorrhagic stroke occurred in 77 (2.3%) participants and ischemic stroke occurred in 141 (4.2%). UCR categories did not show a clear association with increased risk for any type of stroke after adjustment for both age and sex as well as potential confounding factors.

### Cancer death

Cancer death occurred in 107 (3.2%) participants. There was no significant association between UCR categories and incidence risk for cancer death after adjustment for age and sex as well as all potential confounding factors.

### Nonlinear association between UCR and clinical outcomes

**Figure 2:**
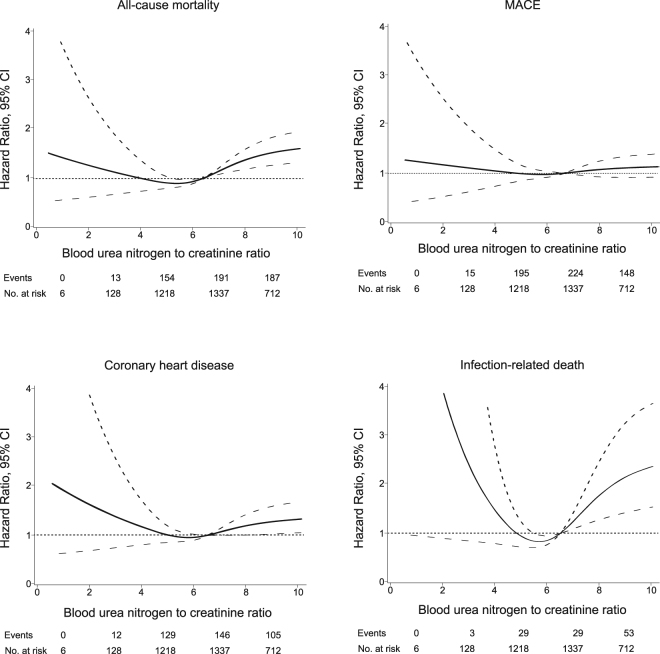
Restricted cubic spline showing the adjusted association between blood urea nitrogen to creatinine ratio (UCR) and all-cause mortality, MACE, coronary heart disease, and infection-related death. MACE: major cardiovascular events. Spline plots were adjusted using Cox regression models. Median value is the reference standard. The Cox model was adjusted for potential risk factors for cardiovascular outcomes. Dashed lines indicate 95% confidence intervals. Events and numbers at risk are shown between values on the x-axis.

Multivariable restricted cubic spline functions for all outcomes are presented in Fig. 2. Cubic spline function graphs suggested that the influence of UCR level on HR for all-cause and infection-related mortality, and the incidence of CHD tended to increase when UCR levels decreased below approximately 6; HR increased significantly at UCR levels above 6. There were no such associations between UCR level and the development of MACE, stroke, and cancer death (data not shown).

### Predictive ability of UCR on the incidence of all-cause mortality

To assess the prognostic impact of UCR on the future risk for mortality, we compared Harrell C statistics between the model incorporating UCR or another protein catabolic marker, nPCR and that not incorporating these factors. Table 3 shows that adding UCR to the relevant model significantly improved the accuracy of the risk assessment for future incidences of all-cause death (0.7814 to 0.7843; *P* = 0.02), but no significant improvement was obtained by adding nPCR (0.7814 to 0.7821; *P* = 0.23). When UCR was incorporated into the model, the performance of the prediction model increased significantly: the NRI was 0.02 (*P* = 0.04), and the IDI 0.05 (*P* = 0.003), respectively.

**Table 3:** Comparison between predictive abilities of models for all-cause mortality using Harrell C statistics.

	Harrell C statistics (95% CI)	Difference from Model 1	P-value *vs*. Model 1
Model 1	0.7814 (0.7629–0.8003)	—	—
Model 1 + nPCR	0.7821 (0.7634–0.8007)	0.0007	0.23
Model 1 + UCR	0.7843 (0.7658–0.8027)	0.0028	0.02

Model 1 was adjusted for age, sex, dialysis vintage, diabetes, history of cardiovascular disease, pre-dialysis systolic blood pressure, serum levels of albumin, corrected calcium, phosphorus, total cholesterol, and log-transformed C-reactive protein, Kt/V, body mass index, and antihypertensive agent use. Abbreviations: nPCR; normalized protein catabolic rate, UCR; urea nitrogen to creatinine ratio.

### Association between UCR components and risk of mortality and morbidity

To evaluate the relevance between UCR components (BUN and Cr) and worse outcomes, we conducted additional multivariable analysis incorporating these variables separately into the mode. Table 4 shows that the result of additional analysis of adding each component of UCR to the relevant model separately. Serum Cr was significantly associated with the risk of all-cause death (HR 0.86; 95% CI 0.81–0.90), MACE (HR 0.93; 95% CI 0.89–0.97), CHD (HR 0.91; 95% CI 0.85–0.96) and infection-related death (HR 0.83; 95% CI 0.74–0.93), while BUN was significant associated only with infection-related death (HR 1.02; 95% CI 1.00–1.03).

**Table 4:** Association between UCR components and risk of mortality and morbidity.

Model	Blood urea nitrogen	Creatinine
	HR (95% CI)	HR (95% CI)
All-cause death		
Age-and sex-adjusted	1.00 (1.00–1.01)	0.82 (0.78–0.86)*
Multivariable-adjusted^a^	1.05 (1.00–1.01)	0.86 (0.81–0.90)*
MACE		
Age-and sex-adjusted	1.00 (1.00–1.01)	0.90 (0.86–0.94)*
Multivariable-adjusted^a^	1.00 (0.99–1.01)	0.93 (0.89–0.97)*
CHD		
Age-and sex-adjusted	1.01 (1.00–1.01)	0.88 (0.84–0.93)*
Multivariable-adjusted^a^	1.00 (1.00–1.01)	0.91 (0.85–0.96)*
Infection-related death		
Age-and sex-adjusted	1.02 (1.00–1.03)*	0.76 (0.69–0.84)*
Multivariable-adjusted^a^	1.02 (1.00–1.03)*	0.83 (0.74–0.93)*
Hemorrhagic stroke		
Age-and sex-adjusted	1.00 (0.99–1.02)	0.98 (0.88–1.10)
Multivariable-adjusted^a^	1.00 (0.98–1.02)	1.01 (0.89–1.15)
Ischemic stroke		
Age-and sex-adjusted	0.99 (0.98–1.01)	0.93 (0.85–1.02)
Multivariable-adjusted^a^	1.00 (0.98–1.01)	0.99 (0.90–1.09)
Cancer death		
Age-and sex-adjusted	0.98 (0.97–1.00)	0.94 (0.85–1.04)
Multivariable-adjusted^a^	0.99 (0.98–1.01)	0.94 (0.84–1.05)

*P < 0.05. Abbreviations: MACE, major cardiovascular events; CHD, coronary heart disease; HR, hazard ratio; CI, confidence interval. ^a^Adjusted for baseline characteristics (age, sex, dialysis vintage, diabetes, history of cardiovascular disease, pre-dialysis systolic blood pressure, serum levels of albumin, corrected calcium, phosphorus, total cholesterol, and log-transformed C-reactive protein, Kt/V, normalized protein catabolic rate, body mass index, and antihypertensive agent use).

## Discussion

The results of this study showed a clear association between elevated UCR and an increased risk for all-cause mortality, infection-related death, and incidence of CHD in maintenance hemodialysis patients. There was no similar association for stroke risk and cancer mortality. These associations remained unaltered, even after adjusting for all potential confounders for all-cause mortality and cardiovascular events. Our findings suggest that UCR is a simple and useful prognostic marker for estimating the risk for mortality and morbidity in maintenance hemodialysis patients.

A few previous reports have examined the correlation between UCR and dietary protein intake in patients with pre-dialysis and maintenance dialysis^13–15^. Hemodialysis patients cannot excrete a significant amount of nitrogen into the urine because of anuria. Hence, the rate of increase in blood urea nitrogen levels is mainly determined by dietary protein intake and muscle protein catabolism at a given dialysis efficacy. However, the level of serum creatinine exclusively reflects skeletal muscle mass in patients with maintenance hemodialysis^22^. Therefore, a higher UCR may indicate a lower creatinine level resulting from diminished muscle mass in sarcopenia, or insufficient urea removal, which would be related to inadequate dialysis or high catabolic stress (inter-current illness, inflammation, or steroid therapy).

UCR showed a positive correlation with the serum C-reactive protein level and protein catabolic rate, whereas it correlated negatively with nutritional indicators such as serum albumin and BMI. These findings suggest a possible link between elevated UCR level and sarcopenia status caused by inflammation, malnutrition, or hypercatabolism. Only the model incorporating UCR level showed a significant improvement in the accuracy of the risk assessment for all-cause mortality, but nPCR, another protein catabolic marker did not. When each component of UCR was separately incorporated into the model, all-cause death, MACE, CHD, and infection-related death showed a significant relationship only with serum Cr level, while infection mortality were significantly associated with both serum Cr and BUN. We speculate that these findings suggest that CVD risk is strongly influenced by muscle mass and nutritional status reflected by serum Cr, while the onset of infection-related death is closely related to hypercatabolic state manifested by elevated BUN levels. These findings suggest that UCR may be a useful marker for assessing future incidences of unfavorable outcomes associated with PEM and MICS in dialysis patients.

We found a significant association between UCR and the incidence of CHD, all-cause mortality, and infection-related death. Many recent epidemiological studies have reported that PEM and inflammation are closely related to hospitalization and mortality in dialysis patients, especially those with infections and CVD^1,2^. It is known that inflammation and malnutrition are common in patients with chronic kidney disease and end-stage renal disease^23–25^. Several studies have reported that the inflammatory process promotes infiltration of inflammatory cells into the intima of blood vessels, accelerating atherosclerosis of coronary arteries^26,27^. Inflammation directly stimulates adhesion molecules and causes vascular endothelial dysfunction^28,29^. Furthermore, proinflammatory cytokines such as tumor necrosis factor-α have been reported to promote not only protein catabolic processes, but also protein degradation and suppress protein synthesis, resulting in a loss of appetite^30^. It has also been reported that certain nutrients such as glutamine and both omega-3 and omega-6 polyunsaturated fatty acids potentiate the immune response^31^. Therefore, PEM can also be regarded as an inflammatory disorder that attenuates the host immune response and leads to vulnerability against infection.

There was no significant association between UCR and the incidence of stroke and cancer death in the current study. Several previous studies have identified independent predictors of stroke in hemodialysis patients, such as hypertension, older age, diabetes, and atrial fibrillation^32,33^. These findings might indicate that the incidence of stroke in hemodialysis patients is closely associated with age-related arteriosclerosis or hemodynamic shear stress^34^ rather than nutritional status or inflammation. Although both nutrition deficiency and immune dysfunction attributed to uremia could be promoting factors for cancer development^35,36^, the results of the current study do not show a clear association between UCR level and cancer mortality. This may be explained by a competing relationship between cancer death and death from other comorbidities. Briefly, the high prevalence of risk factors among maintenance hemodialysis patients, such as malnutrition, inflammation, hypertension, and diabetes, might be associated with an increased risk for death from CVD or infection before the development of cancer death. As a result, it is presumed that the impact of UCR on cancer mortality would have been weakened in people who died from cancer compared with mortality from other comorbidities.

This study has several limitations. First, the single measurement of UCR could result in potential misclassification of the study participants into different UCR categories. If such a misclassification is present, the association found in this study would be weakened, biasing the results toward the null hypothesis. In addition, further research should be required to clarify the relationship between changes in UCR value and worse outcome. Second, another potential source of error is that we had no information about covariates and medical treatments prescribed over the follow-up period. The lack of this information may have biased our results to some extent. The prognosis of hemodialysis patients is determined by various comorbidities such as malnutrition, chronic inflammation, cognitive dysfunction and vascular access failure. Therefore, it should be noted that it may not be sufficient to assess the risk of unfavorable outcomes using only single markers or value. Third, we could not obtain information on pre-dialysis care. If participants with an elevated UCR had experienced chronic dialysis treatment due to acute kidney injury during pre-dialysis care, it would explain the possible link between higher UCR and worse outcome. Finally, a potential limitation on the generalizability of our results should be noted. The participating facilities may not represent all Japanese dialysis centers. Despite these limitations, we believe that our findings provide useful information that will help towards a better understanding of the influence of UCR on mortality and morbidity in maintenance hemodialysis patients.

In conclusion, this study revealed that a higher UCR level was significantly associated with an increased risk for all-cause mortality, infection-related death and incidence of CHD in hemodialysis patients. Further investigations are necessary to determine whether UCR is a potential therapeutic target to reduce the burden of mortality and morbidity in maintenance hemodialysis patients.
